# Supplementary material for: Characterization of Steroid Metabolic Pathways in Established Human and Mouse Cell Models
Source: Int J Mol Sci. 2025 Oct 6;26(19):9721. doi: 10.3390/ijms26199721 (PMC12525206; doi:10.3390/ijms26199721)
Supplement: Supplementary file 1 [file ijms-26-09721-s001.zip › ijms-3877451-supplementary.pdf]

Article

Characterization of Steroid Metabolic Pathways in Established Human and Mouse Cell Models

Therina du Toit [1,2,3,*], Michael Groessl [4], Emanuele Pignatti [1,2], Amanda C. Swart [3,5] and Christa E. Flück [1,2]

1 Department of BioMedical Research, University of Bern, 3008 Bern, Switzerland
2 Division of Pediatric Endocrinology, Diabetology and Metabolism, Department of Pediatrics, Bern University Hospital, University of Bern, 3010 Bern, Switzerland
3 Department of Biochemistry, Stellenbosch University, Stellenbosch 7600, South Africa
4 Department of Nephrology and Hypertension, Inselspital, Bern University Hospital, University of Bern, 3010 Bern, Switzerland
5 Department of Chemistry and Polymer Science, Stellenbosch University, Stellenbosch 7600, South Africa.
* Correspondence: therina.dutoit@unibe.ch

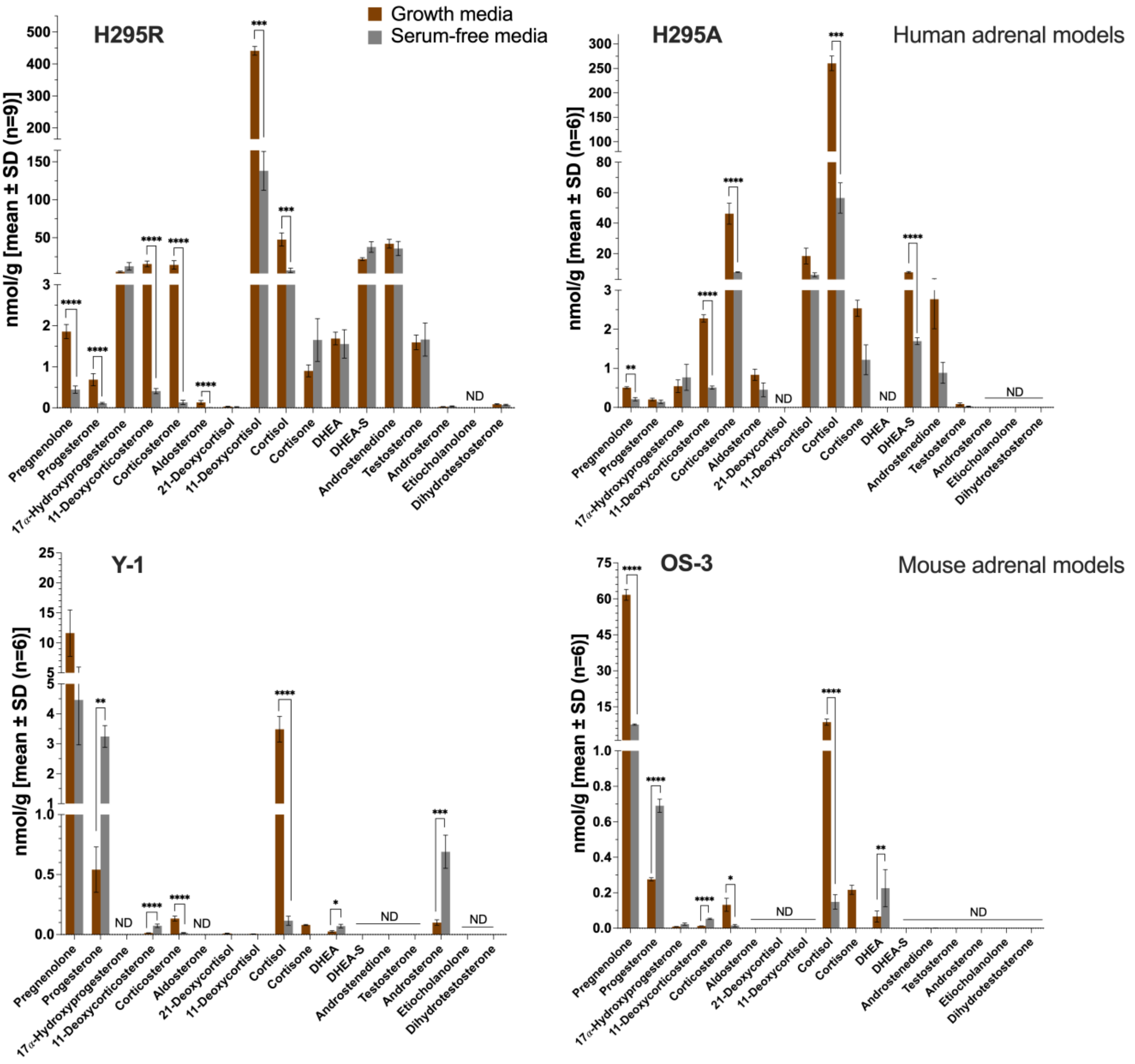

Figure S1. Human and mouse adrenal cell models in complete growth media and serum-free media conditions following a 24 h incubation period. DHEA, dehydroepiandrosterone; DHEA-S, dehydroepiandrosterone sulfate; ND, not detected. *p≤0.05, **p≤0.01, ***p≤0.001, **** p<0.0001.

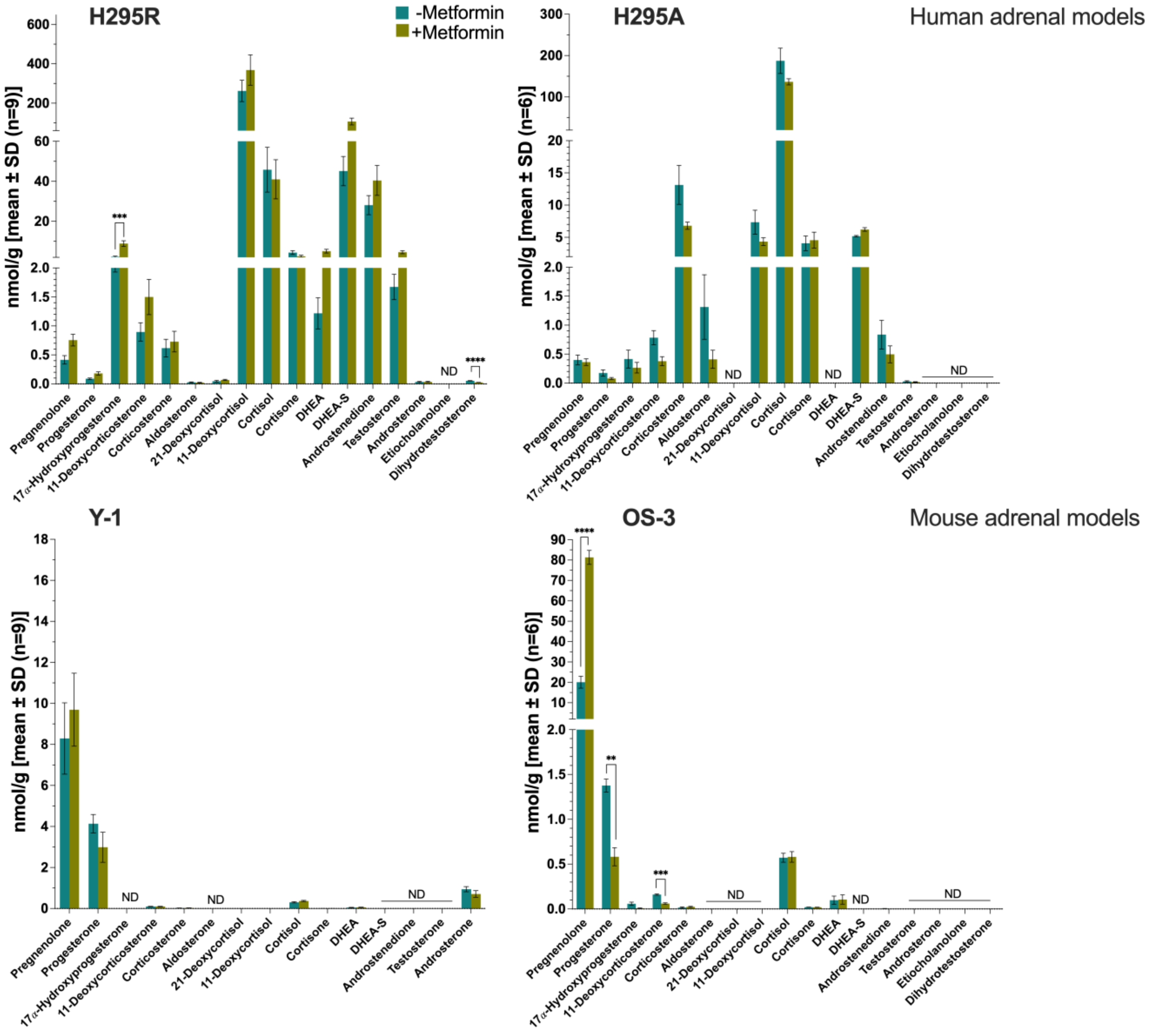

Figure S2. Human and mouse adrenal cell models in serum-free media conditions with and without metformin (10 mM) following a 48 h incubation period. DHEA, dehydroepiandrosterone; DHEA-S, dehydroepiandrosterone sulfate; ND, not detected. **$p \leq 0.01$, ***$p \leq 0.001$, **** $p < 0.0001$.

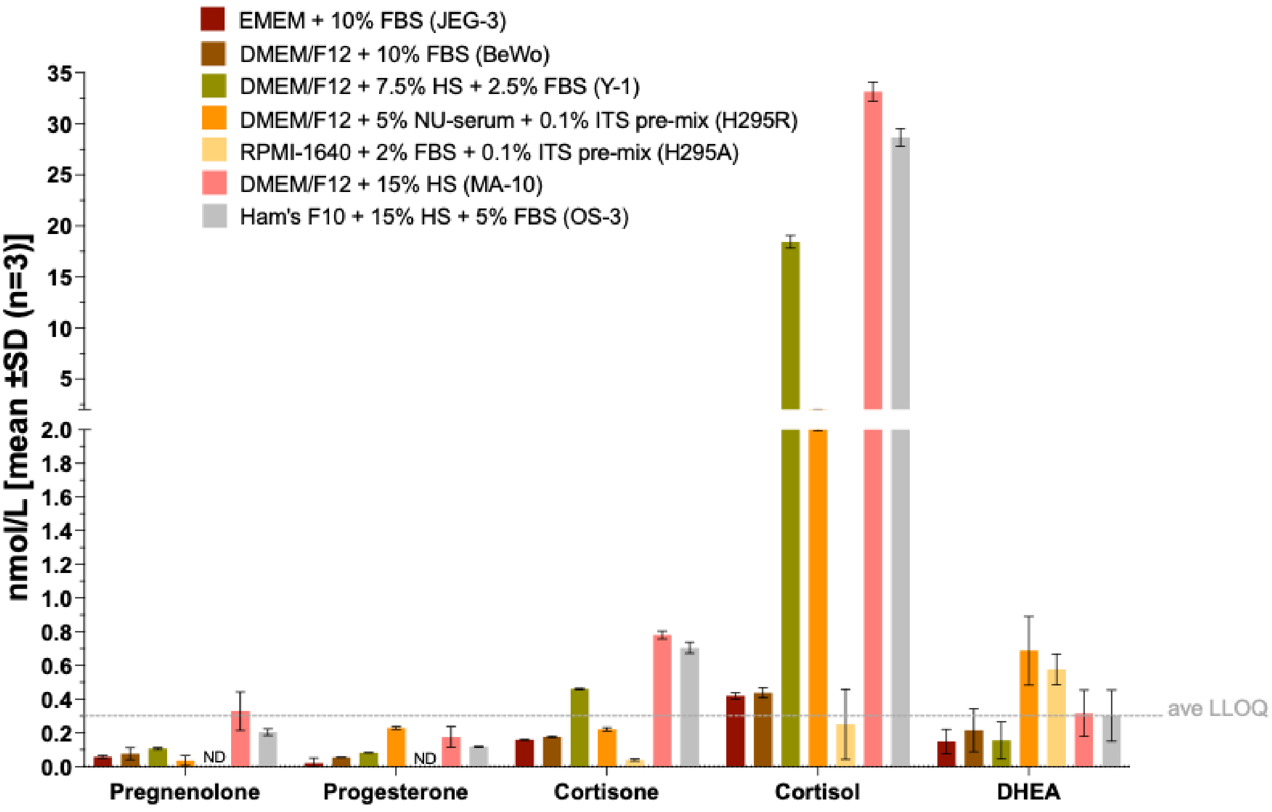

Figure S3. Steroid metabolites quantified in complete growth media (see legend). DHEA, dehydroepiandrosterone; ave. LLOQ, average lower limit of accurate quantification; FBS, fetal bovine serum; HS, horse serum; ITS, insulin-transferrin-selenous acid; ND, not detected.

Table S1. Steroid metabolites measured in the study (LLOQ, lower limit of accurate quantification).

Steroid metabolite	Abbreviation	LLOQ (nmol/L)
Dehydroepiandrosterone	DHEA	0.846
Dehydroepiandrosterone sulfate	DHEA-S	6.252
Androstenedione	-	0.107
Androstenediol	A5	20.00
Testosterone	-	0.105
Dihydrotestosterone	-	0.105
5α-Androstane-3,17-dione	5α-Androstanedione/5αDIONE	6.000
5α-Androstane-3α,17β-diol	5α-Androstanediol/3αDIOL	20.00
11β-Hydroxyandrostenedione	11OH-androstenedione/11OHA4	0.600
11β-Hydroxytestosterone	11OH-testosterone/11OHT	0.150
11-Ketoandrostenedione	11K-androstenedione/11KA4	0.600
11-Ketotestosterone	11K-testosterone/11KT	0.600
11β-Hydroxy-5α-androstane-3,17-dione	11OH-5α-androstanedione/11OH5αDIONE	2.000
11β-Hydroxyandrosterone	11OH-androsterone/11OHAn	2.000
11-Ketoandrosterone	11K-androsterone/11KAn	2.000
Androsterone	-	0.420
Etiocholanolone	-	0.210
Pregnenolone	-	0.771
17α-Hydroxypregnenolone	17OH-pregnenolone/17OHP5	20.00
Progesterone	-	0.476
16α-Hydroxyprogesterone	16OH-progesterone/16OHP4	0.600
6α-Hydroxyprogesterone	6αOH-progesterone/6αOHP4	0.600
6β-Hydroxyprogesterone	6βOH-progesterone/6βOHP4	1.000
11α-Hydroxyprogesterone	11αOH-progesterone/11αOHP4	1.500
11β-Hydroxyprogesterone	11βOH-progesterone/11βOHP4	0.100
11-Ketoprogesterone	11K-progesterone/11KP4	0.200
5α-Pregnanetrione/11-Ketodihydroprogesterone	11K-dihydroprogesterone/11KDHP4	1.000
17α-Hydroxyprogesterone	17OH-progesterone	0.092
21-Deoxycortisol	-	0.088
17α20α-Dihydroxyprogesterone	17,20-diOH-progesterone/17,20diOHP4	0.600
17α-Hydroxypregnanolone (5β-pregnane-3β,17α-diol-20-one)	17OHTHP	10.00
Pregnanetriol	Ptriol	15.00
Pregnanetriolone/11-Ketopregnanetriol	11KPtriol	6.000
20α-Hydroxyprogesterone	20αOH-progesterone/20αOHP4	0.600
20β-Hydroxyprogesterone	20βOH-progesterone/20βOHP4	0.600
5α/β-Dihydroprogesterone	5α/βDHP4	6.000
Pregnanolone (3α,5β-THP)	THP	15.00
5α-Pregnanolone (3α,5α-THP; allopregnanolone)	5αTHP	10.00
5α/β-Pregnan-3β-ol-20-one (3β-hydroxy-5α-THP)	3βTHP	15.00
5α/β-Pregnane-3α, 20α-diol	20OHTHP	100.0
6α-Hydroxypregnanolone	6OHTHP	10.00
5α/β-Pregnane-3β, 20α-diol	20OH-3βTHP	20.00
11-Deoxycorticosterone	-	0.092

Corticosterone	-	0.705
Aldosterone	-	0.085
11-Deoxycortisol	-	0.088
Cortisol	-	0.378
Cortisone	-	0.177

Table S2. Steroidogenic enzyme mRNA expression quantified in cell models based on findings in literature.

Cell model	mRNA expression	References
H295R	*STAR, CYP11A1, HSD3B2, CYP17A1, CYB5, CYP21A2, POR, CYP11B1, CYP11B2, SULT2A1, HSD17B5, HSD17B3*	(1-6)
H295A	*STAR, CYP11A1, HSD3B2, CYP17A1, CYB5, CYP21A2, POR, CYP11B1, SULT2A1, HSD17B5, HSD17B3*	(2, 7)
Y-1	*Star, Cyp11a, Cyp11b1*	(1, 8)
OS-3	-	
BeWo	*CYP11A1, CYP17A1, 11βHSD2, AKR1C3, CYP19A1, SRD5A1, HSD17B1, CYP3A4*	(9-11)
JEG-3	*CYP11A1, HSD3B1, CYP17A1, AKR1C3, CYP19A1, HSD11B1, SRD5A1, HSD17B1, HSD11B2, CYP3A4, HSD17B7, HSD17B12, STS*	(9-13)
MA-10	*Star, Cyp11a1, Hsd3b1, Cyp17a1, Hsd17b1, Hsd17b3, Akr1c14, Srd5a1*	(14, 15)

References

1. Rainey WE, Saner K, Schimmer BP. Adrenocortical cell lines. Mol Cell Endocrinol. 2004;228(1):23-38.
2. Samandari E, Kempná P, Nuoffer JM, Hofer G, Mullis PE, Flück CE. Human adrenal corticocarcinoma NCI-H295R cells produce more androgens than NCI-H295A cells and differ in 3beta-hydroxysteroid dehydrogenase type 2 and 17,20 lyase activities. J Endocrinol. 2007;195(3):459-72.
3. Kempná P, Hirsch A, Hofer G, Mullis PE, Flück CE. Impact of Differential P450c17 Phosphorylation by cAMP Stimulation and by Starvation Conditions on Enzyme Activities and Androgen Production in NCI-H295R Cells. Endocrinology. 2010;151(8):3686-96.
4. Hirsch A, Hahn D, Kempná P, Hofer G, Nuoffer JM, Mullis PE, et al. Metformin inhibits human androgen production by regulating steroidogenic enzymes HSD3B2 and CYP17A1 and complex I activity of the respiratory chain. Endocrinology. 2012;153(9):4354-66.
5. Udhane S, Kempna P, Hofer G, Mullis PE, Flück CE. Differential Regulation of Human 3β-Hydroxysteroid Dehydrogenase Type 2 for Steroid Hormone Biosynthesis by Starvation and Cyclic Amp Stimulation: Studies in the Human Adrenal NCI-H295R Cell Model. PLoS One. 2013;8(7):e68691.
6. Fujii H, Tamamori-Adachi M, Uchida K, Susa T, Nakakura T, Hagiwara H, et al. Marked Cortisol Production by Intracrine ACTH in GIP-Treated Cultured Adrenal Cells in Which the GIP Receptor Was Exogenously Introduced. PLoS One. 2014;9(10):e110543.
7. Wang T, Rainey WE. Human adrenocortical carcinoma cell lines. Mol Cell Endocrinol. 2012;351(1):58-65.
8. Domalik LJ, Chaplin DD, Kirkman MS, Wu RC, Liu WW, Howard TA, et al. Different isozymes of mouse 11 beta-hydroxylase produce mineralocorticoids and glucocorticoids. Mol Endocrinol. 1991;5(12):1853-61.
9. Pavek P, Cerveny L, Svecova L, Brysch M, Libra A, Vrzal R, et al. Examination of Glucocorticoid Receptor α-Mediated Transcriptional Regulation of P-glycoprotein, CYP3A4, and CYP2C9 Genes in Placental Trophoblast Cell Lines. Placenta. 2007;28(10):1004-11.
10. Drwal E, Rak A, Gregoraszczuk E. Co-culture of JEG-3, BeWo and syncBeWo cell lines with adrenal H295R cell line: an alternative model for examining endocrine and metabolic properties of the fetoplacental unit. Cytotechnology. 2018;70(1):285-97.
11. Karahoda R, Kallol S, Groessl M, Ontsouka E, Anderle P, Fluck C, et al. Revisiting Steroidogenic Pathways in the Human Placenta and Primary Human Trophoblast Cells. Int J Mol Sci. 2021;22(4).
12. Samson M, Labrie F, Luu-The V. Specific estradiol biosynthetic pathway in choriocarcinoma (JEG-3) cell line. J Steroid Biochem Mol Biol. 2009;116(3-5):154-9.
13. Kruger L, Yue G, Mettu VS, Paquette A, Sathyanarayana S, Prasad B. Differential proteomics analysis of JEG-3 and JAR placental cell models and the effect of androgen treatment. J Steroid Biochem Mol Biol. 2022;222:106138.
14. Roelofs MJ, van den Berg M, Bovee TF, Piersma AH, van Duursen MB. Structural bisphenol analogues differentially target steroidogenesis in murine MA-10 Leydig cells as well as the glucocorticoid receptor. Toxicology. 2015;329:10-20.
15. Engeli RT, Fürstenberger C, Kratschmar DV, Odermatt A. Currently available murine Leydig cell lines can be applied to study early steps of steroidogenesis but not testosterone synthesis. Heliyon. 2018;4(2).
